# Supplementary material for: Virtual reality interactions via a user-generic ultrasound human-machine interface for wrist and hand tracking
Source: Nat Commun. 2025 Dec 11;16:11062. doi: 10.1038/s41467-025-66001-6 (PMC12699032; doi:10.1038/s41467-025-66001-6)
Supplement: Supplementary file 1 — Supplementary Information [file 41467_2025_66001_MOESM1_ESM.pdf]

Supplementary Information for Virtual Reality Interactions via a User-Generic Ultrasound Human-Machine Interface for Wrist and Hand Tracking

Authors.

Bruno Grandi Sgambato, Bálint K. Hodossy, Deren Yusuf Barsakcioglu, Xingchen Yang, Anette Jakob, Marc Fournelle, Meng-Xing Tang, Dario Farina

Supplementary Discussion 1. Bracelet and Transducer Characterization

The 32 single-element transducers with 1 MHz centre frequency and 65% bandwidth were custom designed for our application. Transducer manufacture started with the realization of a 1 MHz piezocomposite in a dice and fill process from a PZT 5H material processed to the needed thickness for obtaining a 1 MHz centre frequency. The piezocomposite material was then applied on a polyurethane foam backing. Next, a matching layer was added for optimization of the transducer bandwidth. In a last step, the acoustic block was integrated into a 3D printed cylindrical housing, contacting was realized with a twisted pair cable and a thin protective polyurethane layer was applied on the aperture.

Transducer characterization was performed with a calibrated type S hydrophone (RP acoustics, Leutenbach, Germany). XZ sound field pressure maps were measured in a water chamber at 1.1 MHz (Figure S1A-B) and 0.8 MHz (Figure S1C-D). Homogeneity between the 32 elements was tested with a 21 % standard deviation in element sensitivity (Figure S1E). Pressure was measured (at 0.8 MHz with 10 burst pulses) at the acoustic focus for different driving voltages between 15 V and 75 V (with 5 V steps). Voltage was controlled at the acquisition system and could vary slightly at the transducer level. The maximum effective pressure varied between approximately 42 kPa and 130 kPa. Figure S2A shows the values converted to Mechanical Index (MI) in water (with no derating for soft tissue). Transducers were only characterized without the silicone coupling layer.

We experimented with multiple layers of thickness to determine the ideal thickness of the silicone coupling layer in front of the sensors (Figure S2C). To measure the attenuation, small square sections of varying silicone thickness were cured and positioned between the transducer and a reflective interface in a water chamber. The amplitude of the received echo was evaluated for each thickness. These values were compared to the amplitude when no silicone was positioned in the middle. The results revealed that larger silicone thickness can cause substantial attenuation, and that smaller thickness are likely more appropriate. However, we also empirically found that silicone thickness of less than 1 mm was hard to cure properly and very easy to tear. Hence, we chose layers of approximately 1.5 mm. The design of the holders was optimized for comfort. They have a large contact area between the silicone and the skin,

reducing the pressure on the user's skin. The design also included grooves in the plastic and holes at the edges of the holder. This was done to reduce the peeling off between the silicone and holder and allow longer term usage (Figure S2B).

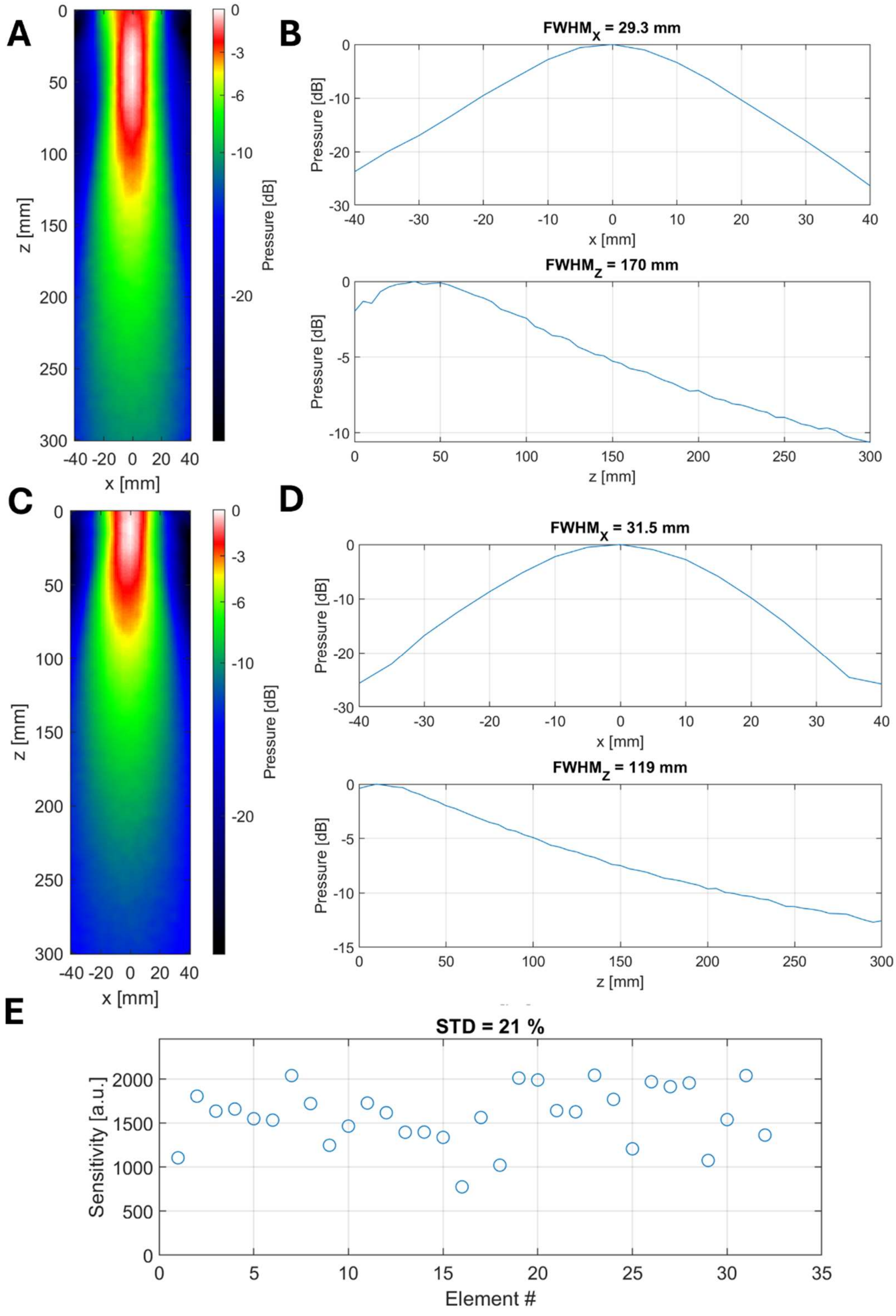

Supplementary Figure S1. Transducer Characterization

(**A-B**) XZ and sound field pressure maps and 2D x and z profiles at the acoustic focus with calculated full-width at half-maximum for 1.1 MHz and (**C-D**) 0.8 MHz. (**E**) Scatter plot of measured element sensitivity for each transducer used with standard deviation of 21% indicated.

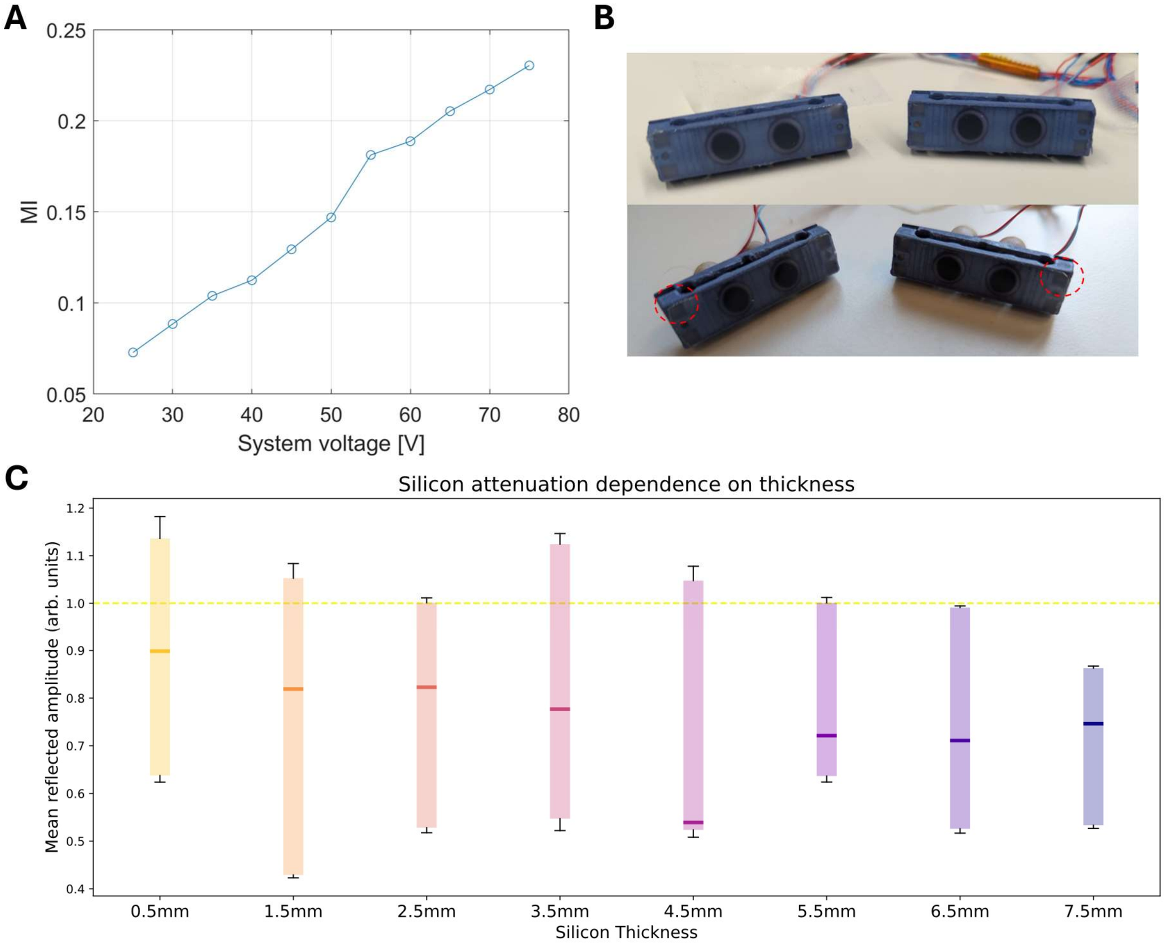

Supplementary Figure S2. Transducer and silicone Characterization

(**A**) Mechanical index (MI) versus voltage for 5 V steps between 15-75 V. Measured in water (without the silicone layer). (**B**) Illustrative picture of two holders with four transducers and the soft-silicone coupling layer. Figure on the top was taken just after silicone curing while figure at the bottom was taken after approximately 4 months of use in multiple participants. Red circles on the figure highlight regions were the silicone layer stared to peel-off from the plastic holder, but no other damage is visible. (**C**) Box and whiskers plot for the relative attenuation of ultrasonic echoes with different silicone thicknesses interposed between sensors and a plastic interface.

Supplementary Discussion 2. Ultrasound Acquisition Processing and Real-Time Implementation

Ultrasound data was acquired using the MoUSE system [2], connected to a host laptop/desktop via USB 3.0. The system works by programming it with a single "Acquisition" sequence that can contain an arbitrary number of "transmission" events. In each event channels can be arbitrarily set to transmit a pulse activation with custom delay. After each transmission event all 32 channels are switched to receive mode for $110\mu s$, with incoming US signals stored in an internal RAM buffer. When the acquisition is completed, the system transfers the saved RF data from its internal RAM to the host computer. The system only starts the new acquisition when the transmission is completed. Therefore, the amount of data acquired in a single acquisition is the main factor controlling the system framerate. Larger or longer acquisitions (e.g., more events, more listening time for each) result in lower framerates. Changes may not be linear due to overheads in the communication.

In our setup we had an average of 11.8 ± 0.5 frames per second or a 377.6 ± 16 Pulse Repetition Frequency (Figure S3A-B). Values were measured over an approximately 2-minute dummy virtual reality control session.

For the offline dataset acquisition data was simply stored in a buffer on the computer RAM and saved after each recording was done. Data was saved in its raw format with no processing. The image formation pipeline was performed after all sessions had concluded and data was stored in a high-performance computer cluster. For the online experiments the image formation pipeline was implemented in python to be performed in real time. Processing steps were performed on the CPU and only the prediction model ran on the host computer GPU. Processing times for key steps in the pipeline are shown in Figure S3C-D. Overall the band-pass filtering and Hilbert enveloping were the slowest steps as they require working on the RF data instead of the much smaller image data. Overall image formation took an average of 60.6 ± 8.5 ms. Offline and online processing pipelines were mostly identical, with the only difference being that the downsampling process on the online pipeline was divided into two steps of 10 times downsampling (instead of one of 100 times). The first step was located just before the Hilbert enveloping in the online pipeline in order to speed up computation and consistently guarantee that processing would be done before the new frame was ready. During the online experiments the raw US data was not saved.

For the online experiments these mesurements result in an average latency of approximately 140 ms between US recording and predicition. Due to the continous nature of the predictions delay between movement and predictions was hard to notice during usage.

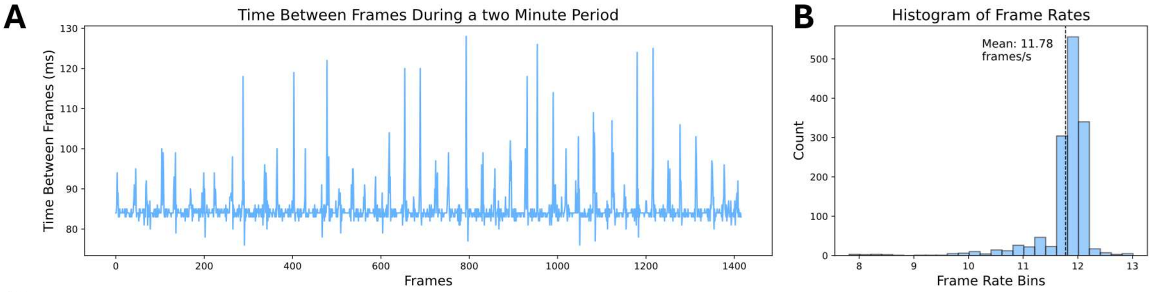

C

Processing step	Mean Time (ms)
Band-Pass Filter	$17.1 \pm 1.8\ ms$
Hilbert Envelop	$34.9 \pm 2.7\ ms$
Model Prediction	$6.8 \pm 7.7\ ms$
Others	$1.8 \pm 0.7\ ms$

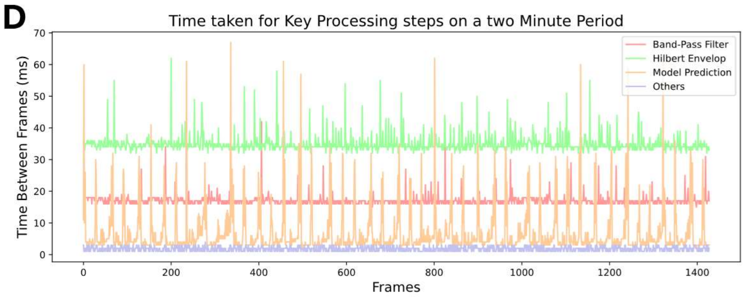

Supplementary Figure S3. Frame-Rate and Online Processing Time Quantification
(**A**) Time between frames (in milliseconds) during an approximately two-minute dummy online experiment. (**B**) Histogram of framerates of the same acquisition, in bins of 0.2 frames. (**C**) Table of average ($\pm$ standard deviation) processing times for key steps on the real-time processing pipeline. Values measured over a second approximately two-minute dummy online experiment. Other includes the sum of the time taken for other processing steps (e.g., array reshaping, downsampling, log-compression, dynamic-range adjustment, pytorch pipeline). (**D**) Time between frames for the same processing steps of table (**C**).

Supplementary Discussion 3. Methodology for Single-Participant Models Evaluation

Figure 3A includes the evaluation of single participant models with the training/testing splits described in the methods section. Distribution of R^2 performance values for the three models were compared and all were statistically significantly different between each other p < 0.0001. For the multi-receive model across all five groups (Figure 3B), the distributions were also all significantly higher than the single-receive models (p < 0.0001). Lastly, for Figure 3C positions 1 and 6 showed significantly higher RMSE distributions (and therefore lower performance) than the other positions with p < 0.0001. Both positions were also significantly different form each other.

Supplementary Discussion 4. Methodology for Multi-Participant Models Evaluation

Figure 4A presents the results for the cross-session and cross-participant evaluations models with different number of participants in the training dataset. Models were trained

using the proposed referencing methodology. This evaluation was conducted by generating a number of distinct training/testing groups for cross-validation. For the cross-session results, models were first trained with only the first session of each participant and tested on the second session (categorized as 1 in the y axis). This resulted in 10 different training/testing scenarios. Subsequent models were trained by adding both sessions of a randomly selected new participant to the training dataset while still testing only on the unseen second session of the first selected participant (categorized as 2, 3, ... in the y axis). This also generated 10 scenarios for each addition. Distributions for each participant count were statistically compared with distributions with one more participant. For example, one participant was compared with two participants. All comparisons were either not statistically significant ($p > 0.05$) or weakly significant (~0.02 between 2-3 participants and between 6-7 participants). For the cross-participant results, more combinations for cross-validation were possible. First, models were trained with all the data from one participant and tested on the data of all other participants (categorized as 1 in the y axis). This resulted in 10 models with 9 testing scenarios each (for a total of 90 scenarios). For subsequent models, new participants were randomly selected, added to the training set, and removed from the testing set (categorized as 2, 3, ... in the y axis). This always resulted in 10 trained models but a varying number of scenarios per model (eight in case of two participants in the training set, seven in case of three until one in the case of nine). In Figure 4A, the results were calculated per recording tested, as the median R^2 of the four predicted DoFs, while figure 4B shows the median R^2 of the two best performing DoFs. Distributions were compared similarly to the cross-session analysis. All distributions were statistically increasing between 1 and 9 participants ($p < 0.0001$ between 1-2, 2-3, 3-4, 4-5, 8-9, $p = 0.003$ between 5-6, and $p = 0.005$ between 6-7) with the exception of the comparison between 8-9, where models with distributions were not statistically different ($p = 0.02$). Figure S4 and S5 (in RMSE terms) includes the same results but separated for each DoF. By separating each the generalization capabilities for each DoF are clearer. We see that the for the pronation-supination and hand opening-closing cross-participant results are both high and close to the cross-session results. This highlights that for these, the models were able to learn a generalizable representation of these movements that worked robustly across users. For the radial-ulnar deviation DoF, however, even for the model with nine participants median R^2 values were barely above 0. This reinforces what was found in the online experiment that participants had little control over the movements of that DoF. Results presented in Figure 4C were calculated in the same way but also included models not using the proposed neutral referencing training methodology.

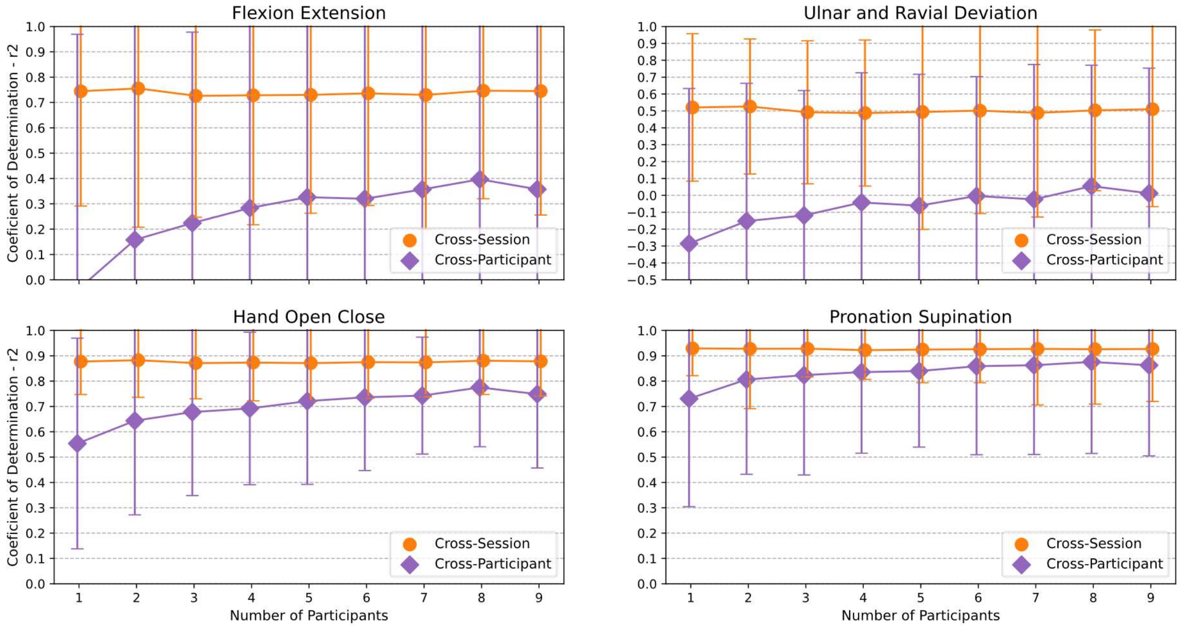

Supplementary Figure S4. Multi-participant Models Evaluation per DoF

Categorical line plot of multi-participant models, from 1 to 9 participants, R^2 performance. Same experiment as Figure 4A but with results for each DoF separately displayed. Error bars represent the distribution standard deviation. Includes results for cross-session (circle, orange) and cross-participant (pentagram, purple) evaluations.

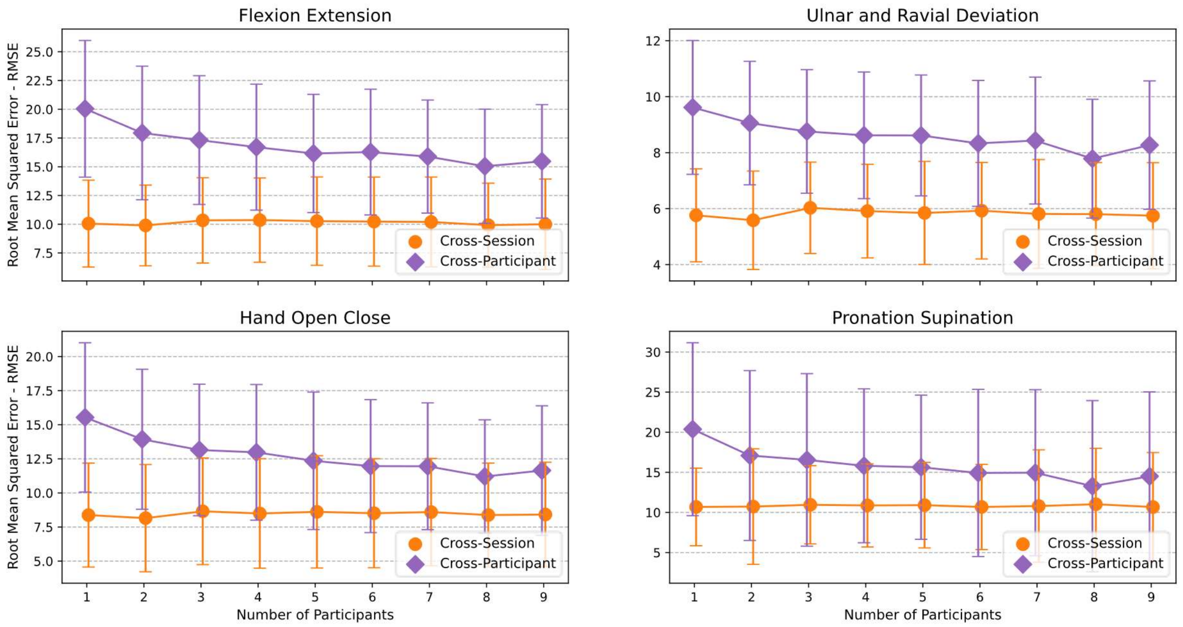

Supplementary Figure S5. Multi-participant Models Evaluation per DoF (RMSE)
Categorical line plot of multi-participant models, from 1 to 9 participants, RMSE performance. Same experiment as Figure 4A but with results for each DoF separately displayed and in terms of RMSE. As the models predicts positions in terms of normalized activation levels, values were converted back to angles before error calculation. Error bars represent the distribution standard deviation. Includes results for cross-session (circle, orange) and cross-participant (pentagram, purple) evaluations.

Supplementary Discussion 5. Artificial Rotations versus Real Rotations

To explore whether artificial bracelet rotations are realistic when compared to the real rotations, we collected data with 16 different bracelet rotations. On one participant (n=1, male, 29 yr), 16 short 5 s recordings without movement were collected. After positioning the bracelet, the position of each transducer holder was marked and numbered on the skin. In the initial position, the first 5 s recording was conducted and then each transducer holder was carefully shifted to the next skin marking clockwise. This was performed 15 times until all the positions were recorded. We calculated the coefficient of correlation between the first 5 s recording, averaged over all frames, both with and without applying a correcting artificial rotation. Figure S6 shows that when corrected, the US images shows a significantly higher coefficient of correlation between them and the original position them when not corrected. This provides the insight that artificial rotations are a reasonable method of providing realistic data augmentation for a dataset.

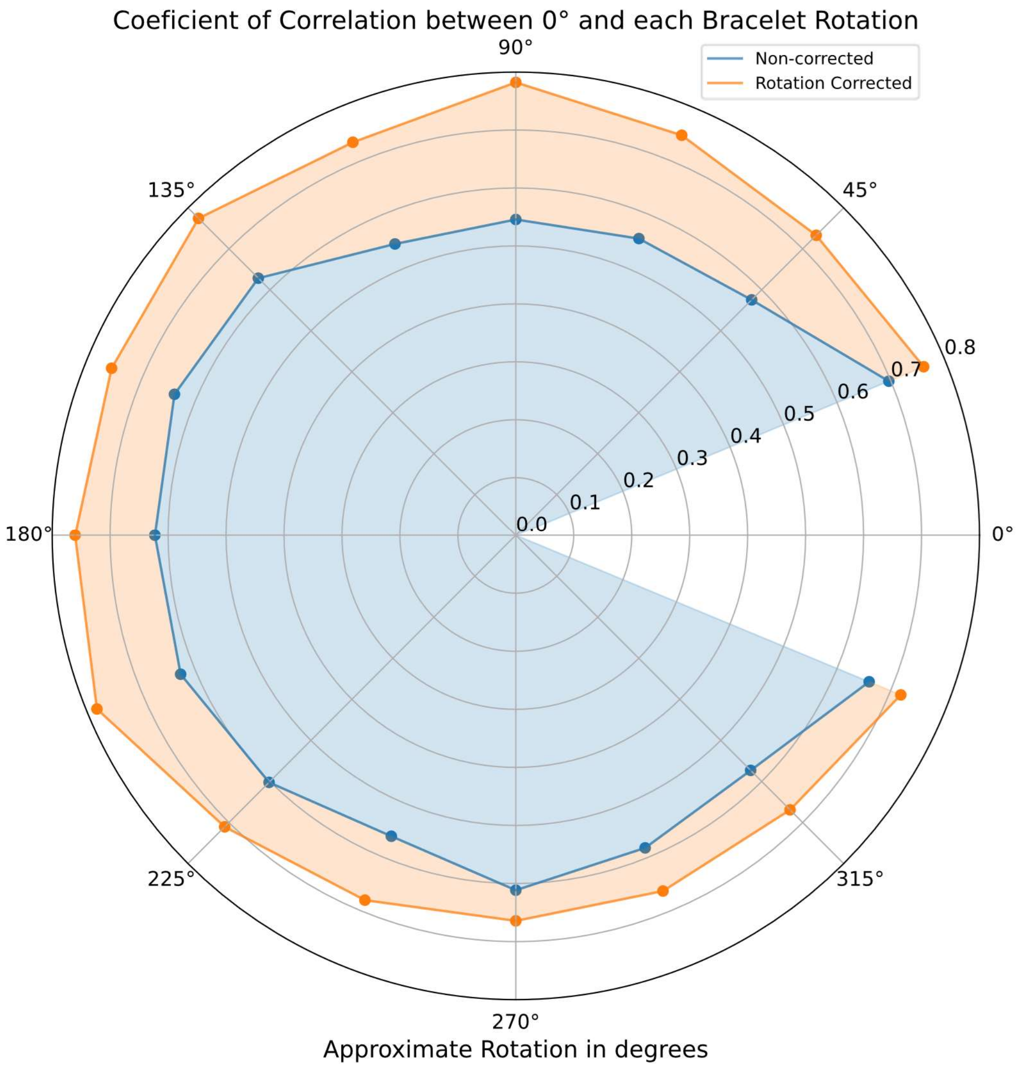

Supplementary Figure S6. Artificial and Real Bracelet Rotations

Polar line and scatter plot showing correlations results between the original 0° bracelet positioning and 15 rotations (angles are approximated). The results are presented for both the original recordings (blue) and artificially rotated recordings (orange). The shaded areas were added for better visualization. The standard deviation was not included as the values were small and not clearly visible.

Supplementary Discussion 6. Data Augmentation

Given the uniqueness of the data generated by the A-mode bracelet system, standard image augmentation methods, developed for computer vision, would likely not be entirely appropriate. Therefore, we proposed custom methods to augment US A-mode data in a manner that would be appropriate. Figure S7 compares the performance of single participant models trained with different augmentation strategies. We tested models with no data augmentation, the Pytorch implementation of TrivialAugment [1], our proposed rotation augmentation, and our proposed rotation augmentation plus blanking and shifting of the image. With the exception of the *Functional* split the use of data augmentation, regardless of the modality, statistically improved performance with $p < 0.0001$ for all paired comparisons between no augmentation and each augmentation method. TrivialAugment has performed surprisingly well specially in the *Position* split with results surpassing the proposed custom augmentations by themselves (but not both combined). This furthers the argument that augmentations are still important in their regularizing effect even it they don't generate realistic domain shift scenarios. The *Rotation* split however, shows that when realistic domain shift scenarios can be recreated, the performance uplift generated by them is extremely relevant promoting a change from approximately 0.3 R^2 to 0.7 R^2 between no augmentation and the artificial rotations. The proposed shift and blacking augmentations improved the performance but were overall the least effective. This was expected as they are a milder version of policies in the TrivialAugment pipeline. While theoretically it is more appropriate to our data, it still showed a weak regularizing effect. Lastly, in all cases, distributions of the combined rotation plus shift and blanking models were statistically higher than other methods ($p < 0.0001$).

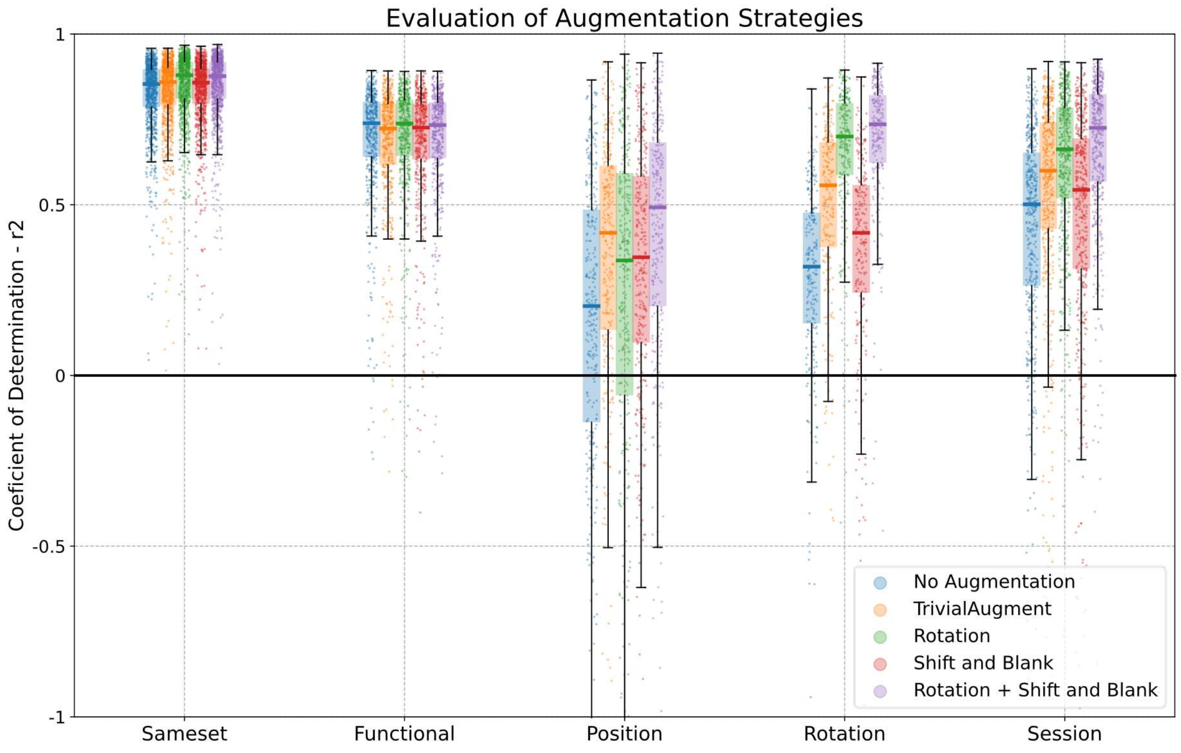

Supplementary Figure S7. Data Augmentation
Box and whiskers plot with scatter points of R^2 for varied data augmentation strategies. Results were calculated for single participant models without the target + rest training method. Scatter values bellow -1 were not included for visualization purposes.

Supplementary Discussion 7. Single-Participant Models Performance per Participant

We compared the results for each participant individually, using the same methodologies to analyze the performance of single participant models against data shifts. Figure S8 shows the median R^2 performance for each recording but separates it by participant. For the *SameSet*, *Functional*, *Rotation* and *Session* splits, the results are fairly clustered with a few outlier participants. The results for the *Position* split, on the other hand, are more variable. In the *Functional* split, the participants 3 and 4 performances were considerably lower than the rest, but that did not necessarily translate to the other splits. The lower performance on the *SameSet* split is the more interesting result. The model should be able to heavily overfit the data and therefore perform ideally in most cases. The lower performance on some participants could point to worse US signal quality due to unsatisfactory coupling or some other source of noise, but visual inspection of the data

showed no clear issues. While we are not certain, we believe this issue is related to subpar optical MOCAP performance and therefore labelling for these participants.

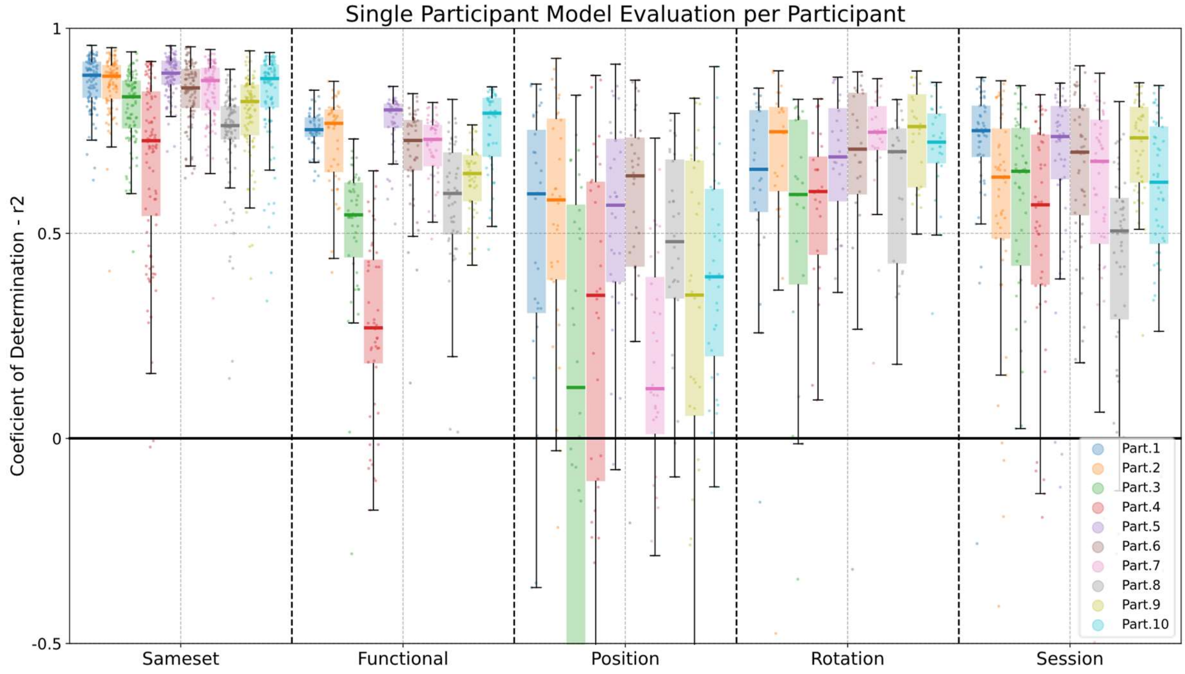

Supplementary Figure S8. Single-Participant Model Performance per Participant
Box and whiskers plot with scatter points of R^2 for with results for each participant independently aggregated. Results were calculated for single participant models without the target + rest training method. Scatter values bellow -1 were not included for visualization purposes.

Supplementary Discussion 8. Performance Distributions to Intra-Participant Shifts

When analyzing single participants models performance to data shifts we can separately plot distributions for each cross-validation fold as a kernel density estimation curve. Figure 3A performs this for the more in-deep cross-validation of the six positions in order to evaluate generalization to positions in-between training samples or beyond training samples. The same can be performed for the cross validation over both recording *Sessions* (Figure S9A) and over the four recording *Positions* (Figure S9B). Unlike the *Position* there was no expectation of different between distributions as there is no reason to believe a specific session would be significantly harder than the other. Similarly, all individual rotations are distributed on a circle and therefore for each cross validation the unseen position is equally distant (and therefore likely equally different) as each other. Our results confirm this expectations as curves in both scenarios are fairly similar.

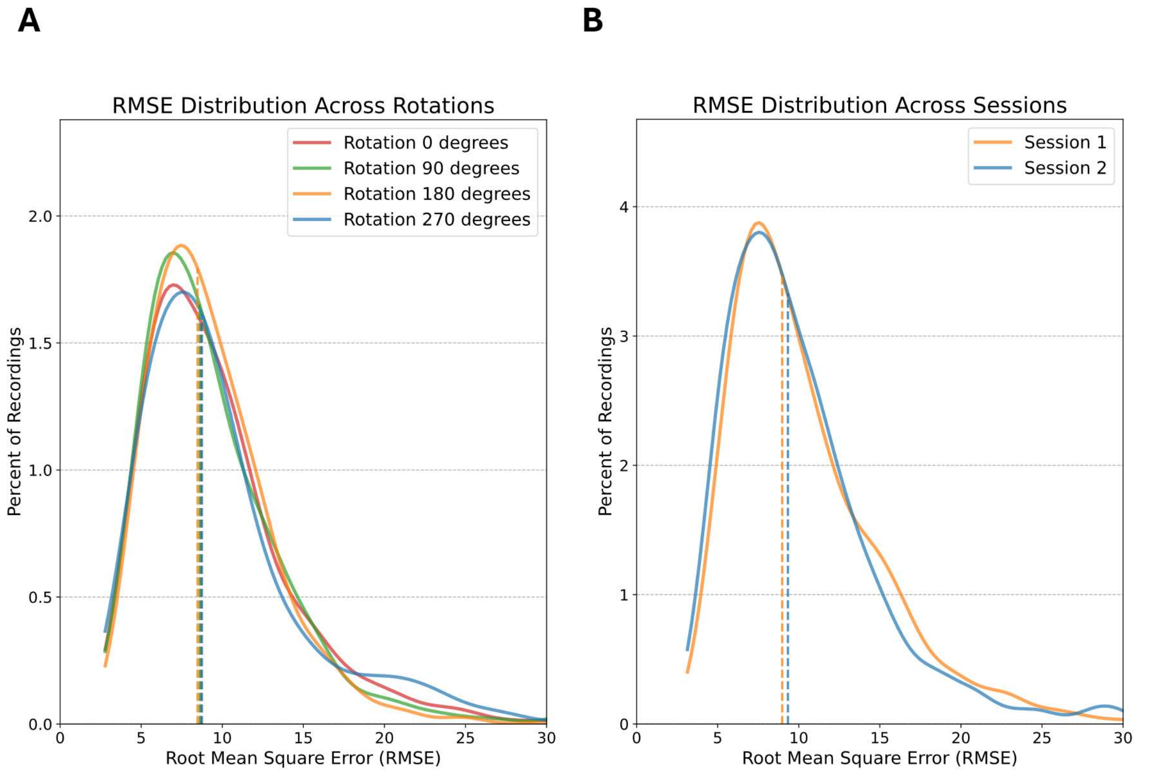

Supplementary Figure S9. Cross-Validation over Rotations and Positions
Kernel density estimations of the distributions of RMSE results for training/testing splits of different *Rotations* (**A**) and *Sessions* (**B**). Doted line marks the median of each distribution.

Supplementary Discussion 9. Correlation between performance over individual DoFs

We questioned whether performances across DoFs were correlated. We tested whether in a single recording a low (high) performance in one DoF correlated with low (high) performance across the other DoFs. This could point towards low performing recordings being a result of unsatisfactory coupling, de-synchronization between US and MOCAP or very unique movements being performed during that specific recording. Figure S10 shows six 2D scatter plots with all combinations of two DoFs included. Each scatter represents two DoF values for one repetition. We calculated the correlation between the scatter points in each graph and plotted the best fitting linear regression curve. In all six cases linear correlations were low showing that there is not any likely linear correlation between high (or low) performance between DoFs in each recording. Visual inspection also does not seem to point to any other obvious consistent relationship between DoFs.

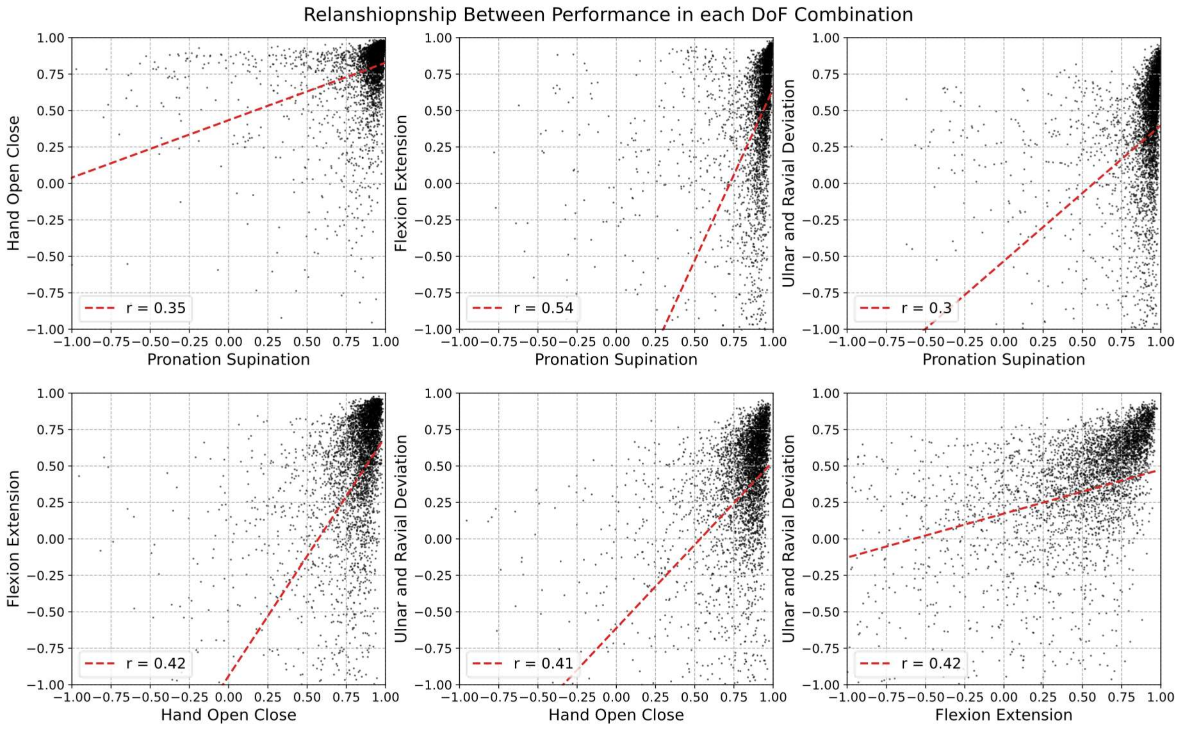

Supplementary Figure S10. Scatter points relationships between performances in each DoF Scatter plots of recording R^2 results for all pairs of DoFs. Each includes the least-squares best fitting linear function for all values with the correlation coefficient as legend.

Supplementary Discussion 10. Effect of prediction of normalized joint pose or joint angles.

In the final models we have converted the joint angles calculated by the MOCAP data and the inverse kinematics models to a normalized “joint activation” (or joint pose) measure. This was done as different people can have reasonable varied ranges of motion. In our recordings we also saw that even the same participants could have their ranges vary between sessions, likely due to slight variations on maker positioning and different effort levels by each participant to reach its "maximum" activations.

Figure S11 explores the performance differences between training single-participant models on the normalized “joint activations” or on the raw joint angles. Surprisingly there is a performance difference even in single participant models. Significant performance differences improvement ($p < 0.0001$) is seen for the models trained on normalized activations for all cross-validations, except the Rotation group. As expected, the most significant change is seen in the cross-session evaluation.

It’s important to highlight that this normalization approach is appropriate for the use case explored (VR control) as it does not need absolute angle values as outputs of the model (the normalized activations can be scaled to any arbitrary range used by the interface).

Therefore, any new user can use the pretrained models and control the twin limb appropriately without any calibration. However, for other applications (e.g., biomechanics research) where the desired output of the model may be the absolute joint angles a calibration to individual users' range of motion would be needed.

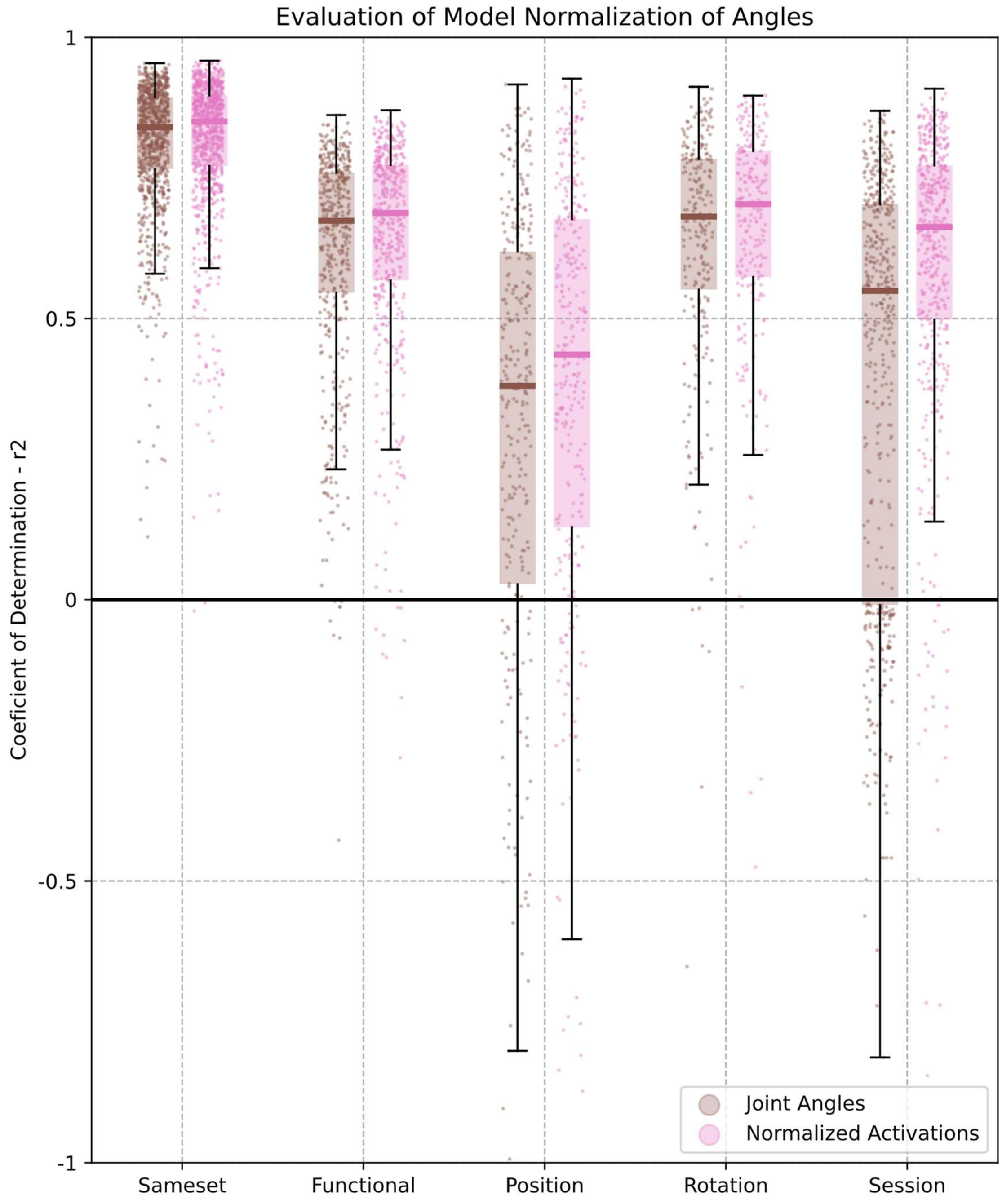

Supplementary Figure S11. Single-Participant Model Performance by using joint angles or normalized joint activations.
Box and whiskers plot with scatter points of R^2 with results for models trained on the raw joint angles and on the session normalized joint activations. Results were calculated for single participant models without the target + rest training method. Scatter values bellow -1 were not included for visualization purposes

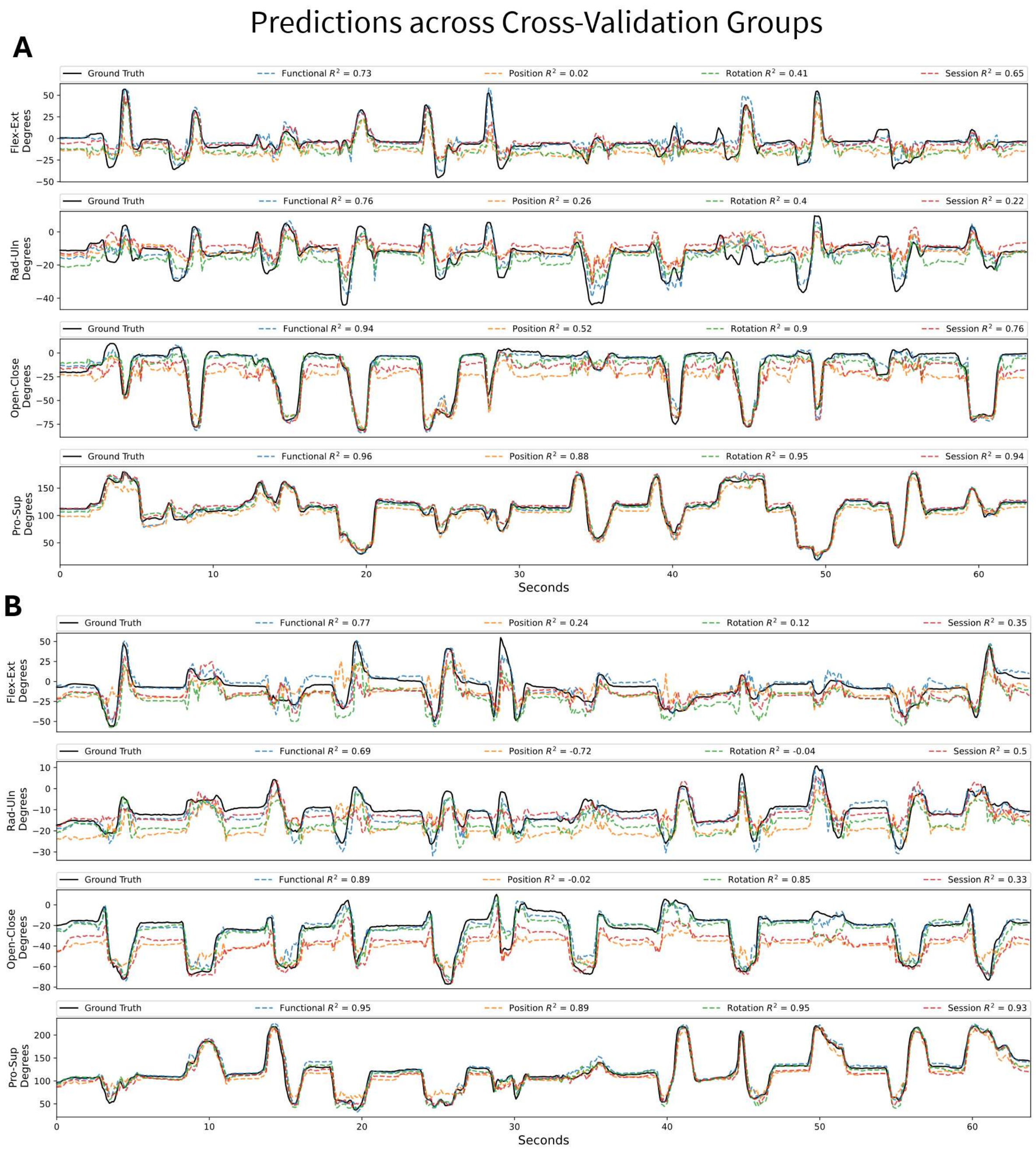

Supplementary Figure S12. Example of Angle Predictions of Single Participant Models across Cross-Validation Groups

Example of predictions for the four DoFs on the same unseen recording depending on the cross-validation group used as training set. The same evaluations as the ones summarized on Figure 3 were used. The model used was the Conv + Aug model. In both examples it's clear that the low R^2 values for the *Position* group are mainly caused by consistent offsets in prediction. The same is also true for the *Session* group in some cases. (**A**) The unseen recording shown is from participant 2, position 3, rotation 4, session 2 wrist movements. (**B**) The unseen recording shown is from participant 4, position 3, rotation 4, session 2 wrist movements. Predictions were made into normalized values and approximately converted back to angles based on the range of movement calculated for the participant.

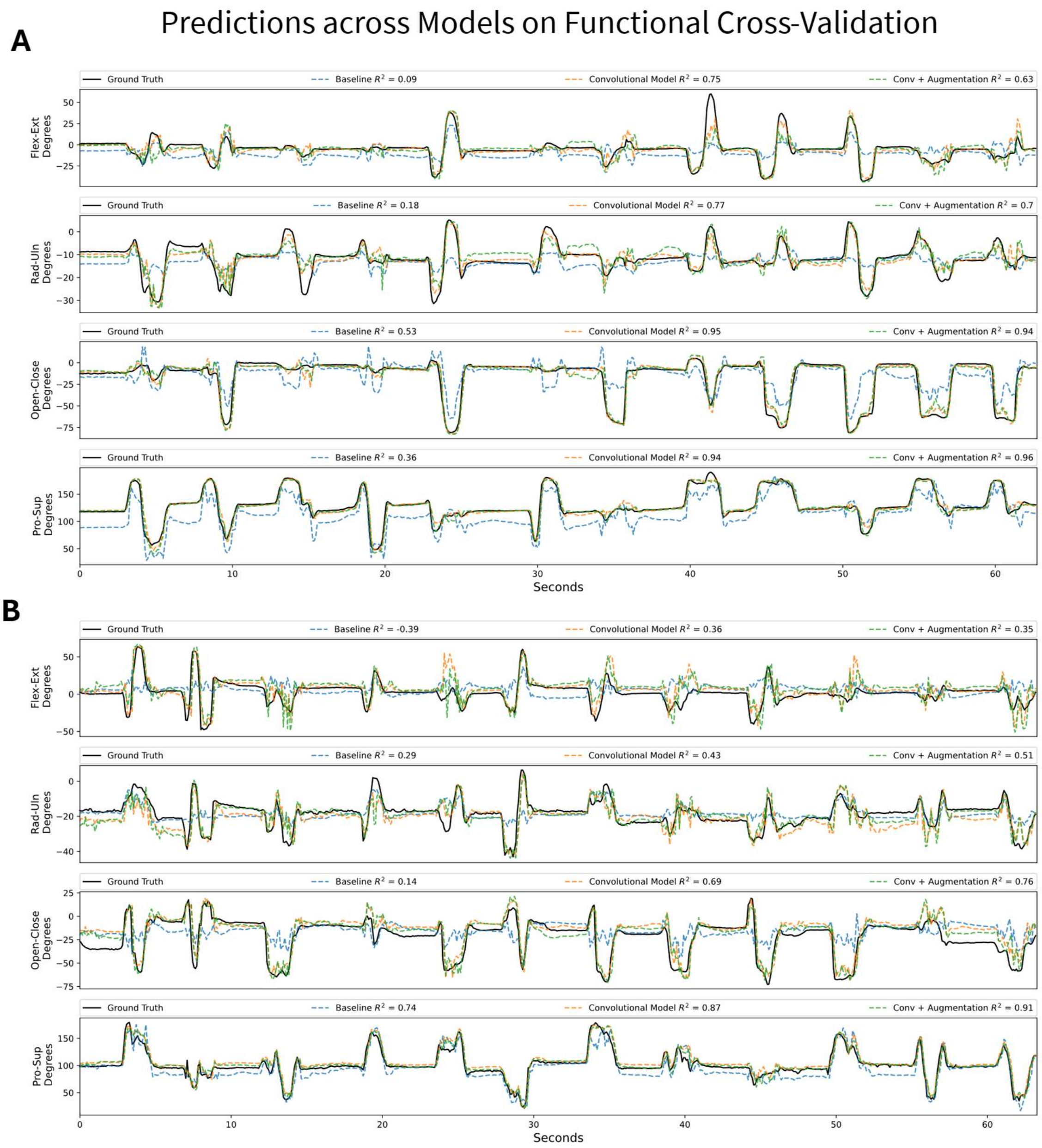

Supplementary Figure S13. Example of Angle Predictions for the Different Single Participant Models on the Functional Cross-Validation Group

Example of predictions, of different models, for the four DoFs on the same unseen recording based on training on the *Functional* cross-validation group. The same evaluations as the ones summarized on Figure 3 were used. On example (**A**) the baseline model is seen show a strong offset in predictions of Pro-Sup that was correct by the Convolutional models, while on example (**B**) all three models performed more similarly. Interestingly, for the hand Open-Close DoF on example (**B**) the baseline model seemed to have not been able to track any of the cases of finger hyper-extension (angles bigger than 0), while on example (**A**) it predicted hyper-extensions when there were none. In both cases the Convolutional models performed well. In some cases, such as Flex-Ext of example (**B**), while predictions mostly followed the movements none of the models had particularly strong R^2 performance. (**A**) The unseen recording shown is from participant 1, position 2, rotation 1, session 2 functional movements. (**B**) The unseen recording shown is from participant 2, position 3, rotation 2, session 2 functional movements. Predictions were made into normalized values and approximately converted back to angles based on the range of movement calculated for the participant.

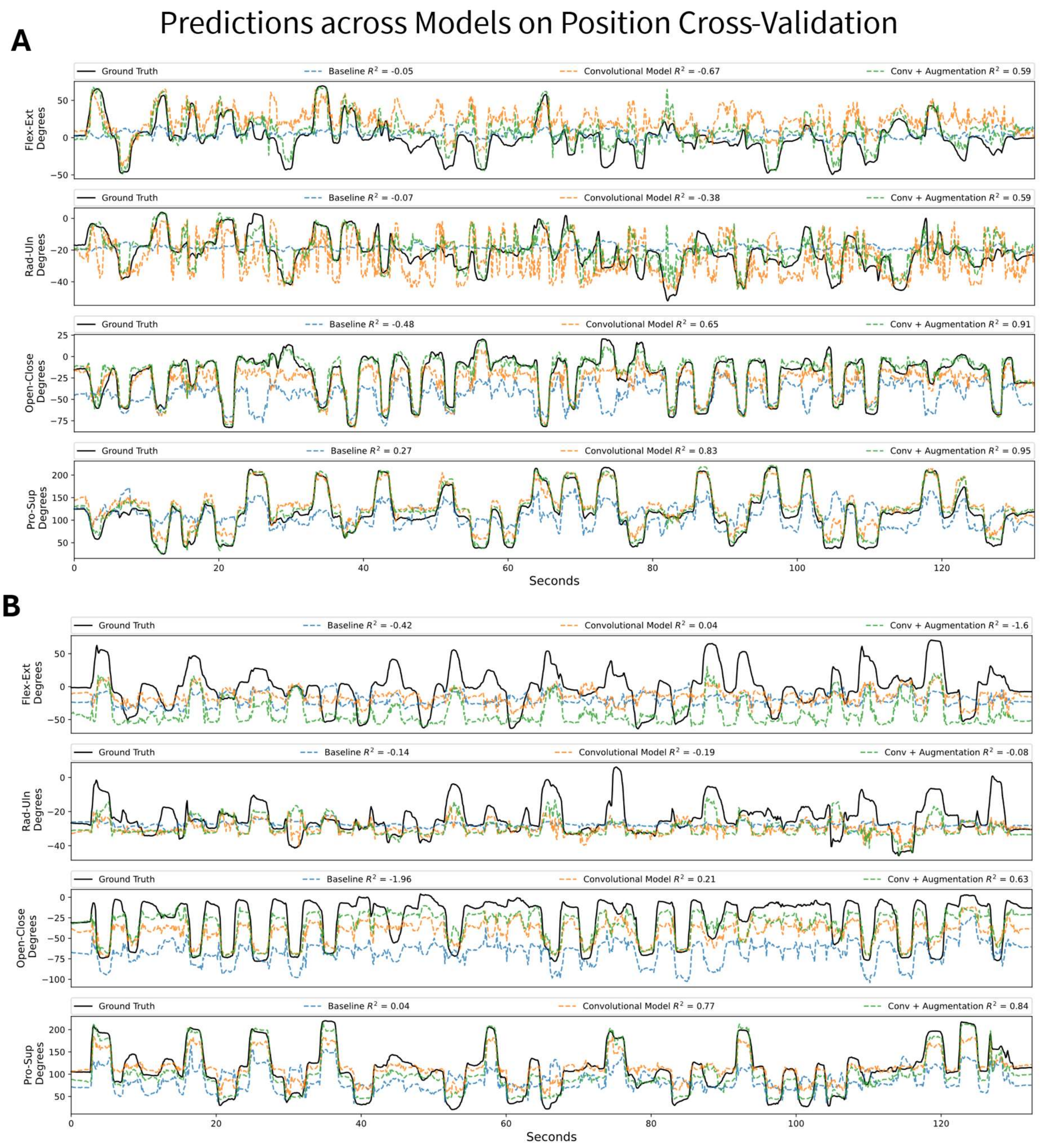

Supplementary Figure S14. Example of Angle Predictions for the Different Single Participant Models on the Position Cross-Validation Group

Example of predictions, of different models, for the four DoFs on the same unseen recording based on training on the *Position* cross-validation group. The same evaluations as the ones summarized on Figure 3 were used. For the Position group the offset effect on predictions is very noticeable and one of the main reasons for poor R^2 performance on the baseline model, but normally corrected by the Convolutional models, this is visible on the hand Open-Close and Pro-Sup of both examples. In some situations, the Convolutional models are still not able to correct for the offset properly, visible on the Flex-Ext DoF on example (**B**). (**A**) The unseen recording shown is from participant 6, position 1, rotation 2, session 1 wrist movements. (**B**) The unseen recording shown is from participant 9, position 3, rotation 3, session 1 wrist movements. Predictions were made into normalized values and approximately converted back to angles based on the range of movement calculated for the participant.

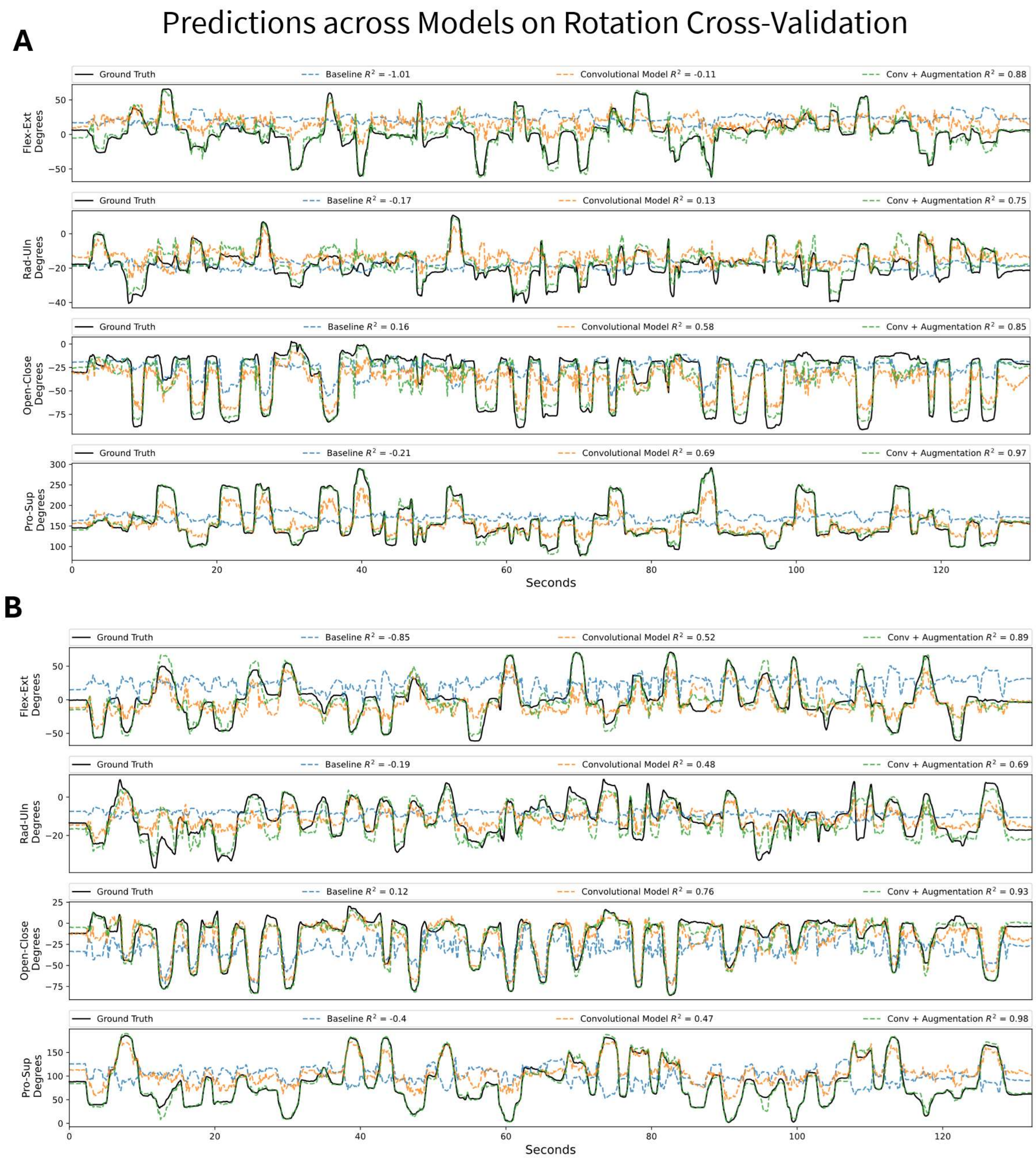

Supplementary Figure S15. Example of Angle Predictions for the Different Single Participant Models on the Rotation Cross-Validation Group

Example of predictions, of different models, for the four DoFs on the same unseen recording based on training on the *Rotation* cross-validation group. The same evaluations as the ones summarized on Figure 3 were used. The *Rotation* group specifically shows the power of the Convolutional model and the augmentation approach. On all DoFs of both examples the baseline performed very poorly, sometimes mostly just predicting values close to the mean (Pro-Sup example (**A**)). The Convolutional model shows marked increase in performance with even more improvements by also augmenting the data, with all R^2 values above 0.69 of the Conv + Aug in both examples. (**A**) The unseen recording shown is from participant 10, position 2, rotation 1, session 1 wrist movements. (**B**) The unseen recording shown is from participant 1, position 1, rotation 3, session 2 wrist movements. Predictions were made into normalized values and approximately converted back to angles based on the range of movement calculated for the participant.

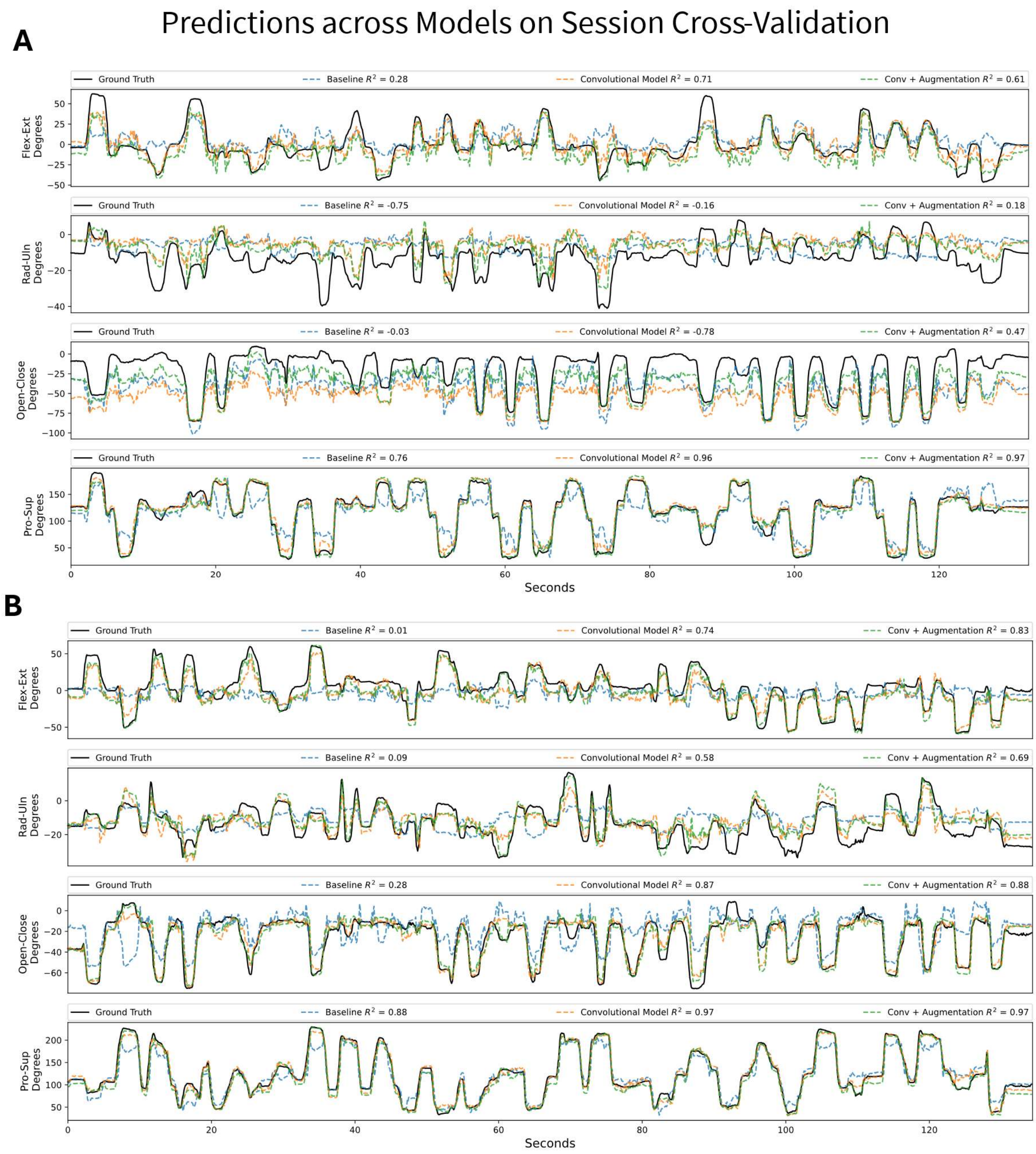

Supplementary Figure S16. Example of Angle Predictions for the Different Single Participant Models on the Session Cross-Validation Group

Example of predictions, of different models, for the four DoFs on the same unseen recording based on training on the *Session* cross-validation group. The same evaluations as the ones summarized on Figure 3 were used. On the Session group, similarly to others, results improved for most DoFs for the Convolutional models. However, a lot of the improvement, in these two examples, looks to be linked to overall better tracking and not a reduction of any prediction offset. This is seem in the Flex-Ext DoF of both examples. Interestingly, on the hand Open-Close DoF of example (**A**) the predictions were worse for the Convolutional model (when compared to the baseline) but recovered by the use of data augmentation. (**A**) The unseen recording shown is from participant 2, position 2, rotation 1, session 2 wrist movements. (**B**) The unseen recording shown is from participant 5, position 2, rotation 1, session 2 wrist movements. Predictions were made into normalized values and approximately converted back to angles based on the range of movement calculated for the participant.

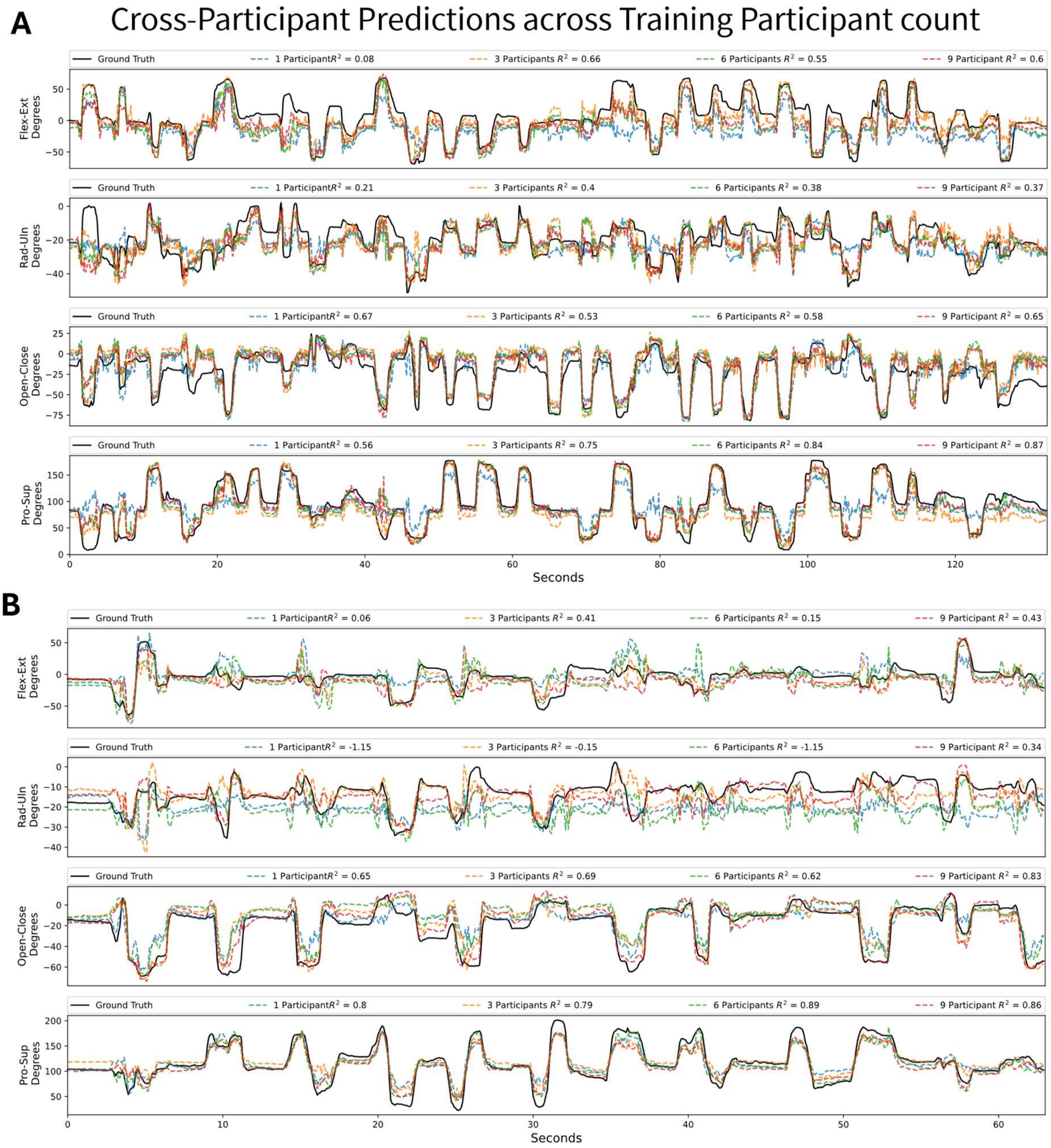

Supplementary Figure S17. Example of Angle Predictions for Multi-Participant Models on the Cross-Participant Validation with Varying Amounts of Participants on the Training Set
Example of cross-participant predictions for the four DoFs on the same unseen recording based on training with different amounts of participants on the training dataset. The same evaluations as the ones summarized on Figure 4 were used. The models on this figure used the referencing strategy. Overall, noticeable grow in performance from increased amount of participants in the training dataset is seen. On the Pro-Sup DoF of example (**A**) the increase from having more than 1 participant, for example, is very notable. Some other cases, such as the Rad-Uln and hand Open-Close DoFs of example (**A**), show little improvement from having more examples. (**A**) The unseen recording shown is from participant 3, position 1, rotation 3, session 2 wrist movements. (**B**) The unseen recording shown is from participant 8, position 2, rotation 1, session 2 functional movements. Predictions were made into normalized values and approximately converted back to angles based on the range of movement calculated for the participant.

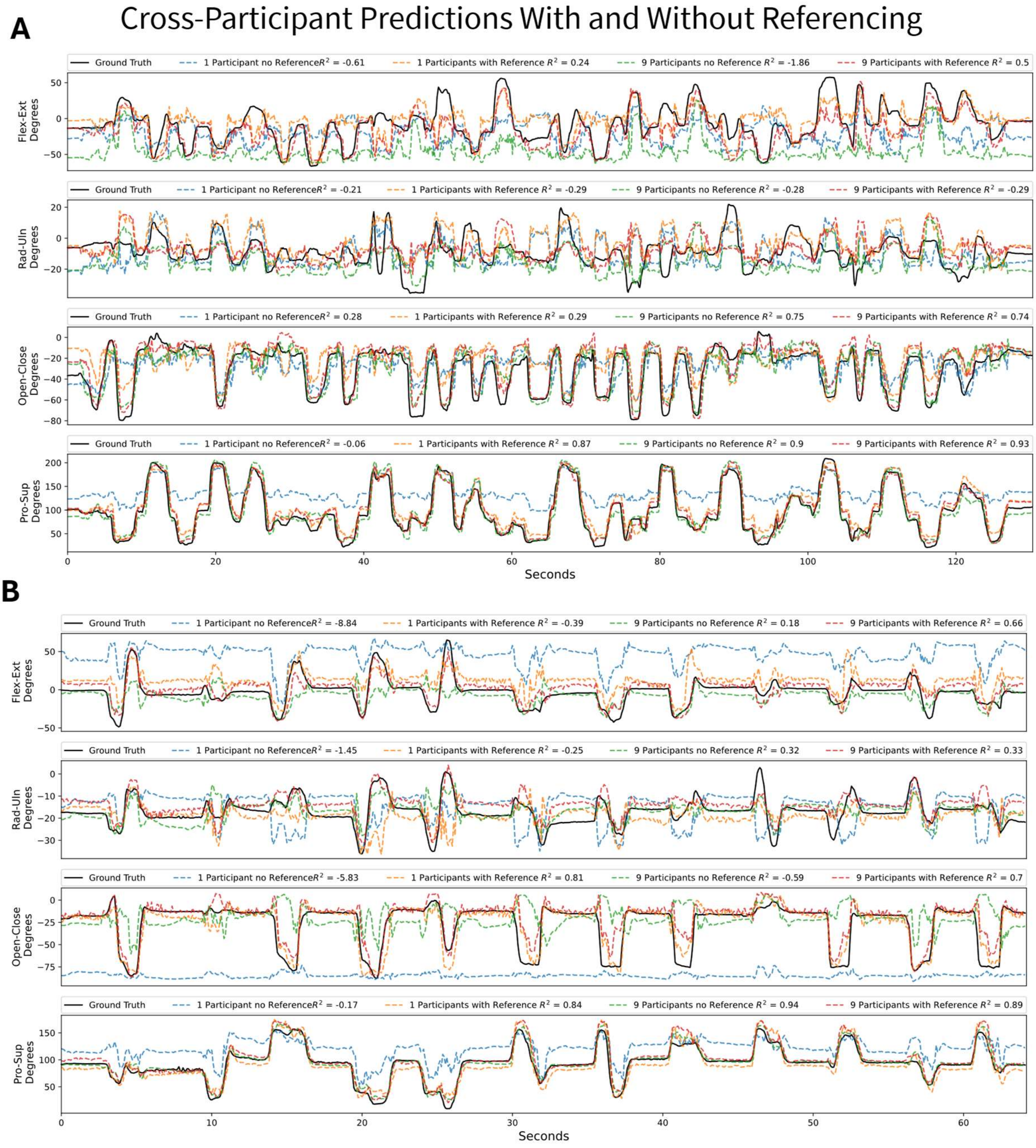

Supplementary Figure S18. Example of Angle Predictions for Multi-Participant Models on the Cross-Participant Validation With and Without Referencing

Example of cross-participant predictions for the four DoFs on the same unseen recording based on training with and without the referencing strategy on either 1 or 9 participants on the training dataset. The same evaluations as the ones summarized on Figure 4 were used. As seen, the introduction of the referencing strategy has very notable implications on performance. Results for the hand Open-Close DoF on example (**B**) and for the Pro-Sup DoF on example (**A**), for example, were completely unusable, but showed massive improvements by the introduction of referencing. In both cases the improvements were similar to the ones achieved by the introduction of 9 new participants on the training set. On the hand Open-Close DoF of example (**A**), on the other hand, the referencing strategy showed little improvement while the main responsible for the final performance was the addition of more participants on the training set. Lastly, on the Rad-Uln DoF of example (**A**), no performance increases were seen and results remained low. (**A**) The unseen recording shown is from participant 5, position 3, rotation 3,

session 1 wrist movements. (**B**) The unseen recording shown is from participant 7, position 2, rotation 1, session 2 functional movements. Predictions were made into normalized values and approximately converted back to angles based on the range of movement calculated for the participant.

Supplementary References

[1] S. G. Muller and F. Hutter, 'TrivialAugment: Tuning-free Yet State-of-the-Art Data Augmentation', in 2021 IEEE/CVF International Conference on Computer Vision (ICCV), 2021, pp. 754–762.

[2] M. Fournelle, T. Gr ̈un, D. Speicher, S. Weber, M. Yilmaz, D. Sch ̈ob, A. Miernik, G. Reis, S. Tretbar, and H. Hewener, "Portable ultrasound research system for use in automated bladder monitoring with machine-learning-based segmentation," Sensors, vol. 21, p. 6481, 2021.
