# Supplementary material for: Virtual reality interactions via a user-generic ultrasound human-machine interface for wrist and hand tracking
Source: Nat Commun. 2025 Dec 11;16:11062. doi: 10.1038/s41467-025-66001-6 (PMC12699032; doi:10.1038/s41467-025-66001-6)
Supplement: Supplementary file 2 — Description of Additional Supplementary Files [file 41467_2025_66001_MOESM2_ESM.pdf]

Description of Additional Supplementary Files

Supplementary Movie 1 briefly illustrates the key points and methodology applied in the study. It also provides video examples of participants controlling the system and performing the interaction tasks in the virtual environment.
