# Supplementary material for: Virtual reality interactions via a user-generic ultrasound human-machine interface for wrist and hand tracking
Source: Nat Commun. 2025 Dec 11;16:11062. doi: 10.1038/s41467-025-66001-6 (PMC12699032; doi:10.1038/s41467-025-66001-6)
Supplement: Supplementary file 4 — Reporting Summary [file 41467_2025_66001_MOESM4_ESM.pdf]

nature portfolio

Corresponding author(s): Dario Farina

Last updated by author(s): Sep 10, 2025

Reporting Summary

Nature Portfolio wishes to improve the reproducibility of the work that we publish. This form provides structure for consistency and transparency in reporting. For further information on Nature Portfolio policies, see our Editorial Policies and the Editorial Policy Checklist.

Statistics

For all statistical analyses, confirm that the following items are present in the figure legend, table legend, main text, or Methods section.

n/a	Confirmed	
☐	☒	The exact sample size (*n*) for each experimental group/condition, given as a discrete number and unit of measurement
☒	☐	A statement on whether measurements were taken from distinct samples or whether the same sample was measured repeatedly
☐	☒	The statistical test(s) used AND whether they are one- or two-sided<br>*Only common tests should be described solely by name; describe more complex techniques in the Methods section.*
☐	☒	A description of all covariates tested
☐	☒	A description of any assumptions or corrections, such as tests of normality and adjustment for multiple comparisons
☐	☒	A full description of the statistical parameters including central tendency (e.g. means) or other basic estimates (e.g. regression coefficient) AND variation (e.g. standard deviation) or associated estimates of uncertainty (e.g. confidence intervals)
☐	☒	For null hypothesis testing, the test statistic (e.g. *F*, *t*, *r*) with confidence intervals, effect sizes, degrees of freedom and *P* value noted<br>*Give P values as exact values whenever suitable.*
☒	☐	For Bayesian analysis, information on the choice of priors and Markov chain Monte Carlo settings
☒	☐	For hierarchical and complex designs, identification of the appropriate level for tests and full reporting of outcomes
☒	☐	Estimates of effect sizes (e.g. Cohen's *d*, Pearson's *r*), indicating how they were calculated

Our web collection on statistics for biologists contains articles on many of the points above.

Software and code

Policy information about availability of computer code

Data collection	Ultrasound data was collected with the MoUSE system from Fraunhofer IBMT via the API made for it. Motion capture data was collected with a Vicon system using the commercial Vicon Nexus (1.6) software. Online validation was performed on and virtual environment created in Unity (2022.3.2f1). Videos were recorded with standard web cameras using OBStudio (30.2.3).
Data analysis	Ultrasound data was processed and analysed by custom code prepared for this work. Code uses varied public Python libraries (e.g., NumPy, pandas, Pytorch) with and accompanying descriptions on how to setup an environment with the specific versions used. Code is available at: github.com/BrunoSgambato/VR-HMI-US/. Optical motion capture data was initially processed, cleaned, and analyzed using Vicon Nexus (1.6) and the Python API available for it.

For manuscripts utilizing custom algorithms or software that are central to the research but not yet described in published literature, software must be made available to editors and reviewers. We strongly encourage code deposition in a community repository (e.g. GitHub). See the Nature Portfolio guidelines for submitting code & software for further information.

Data

Policy information about availability of data

All manuscripts must include a data availability statement. This statement should provide the following information, where applicable:

- Accession codes, unique identifiers, or web links for publicly available datasets
- A description of any restrictions on data availability
- For clinical datasets or third party data, please ensure that the statement adheres to our policy

All processed data generated in this study and shown in both main text and supplementary material have been deposited on GitHub (github.com/BrunoSgambato/VR-HMI-US/paper) under a CC BY-NC 4.0 license. The raw ultrasound, motion capture data, and videos are not available due to its large size. The processed ultrasound and motion capture data for one volunteer participant has been deposited on Zenodo (doi.org/10.5281/zenodo.17296286) under CC BY-NC 4.0 license

Research involving human participants, their data, or biological material

Policy information about studies with human participants or human data. See also policy information about sex, gender (identity/presentation), and sexual orientation and race, ethnicity and racism.

Reporting on sex and gender	Participant population included male and female sexes. Data on sex was self-reported. No data on gender was collected. To the best of the authors knowledge, no research so far has shown that results for ultrasound-based control methods are influenced by participant's sex. Therefore, no considerations to it were made during recruitment and analysis. Reported participant sexes are included as a total for each reported sex. Sex and other characteristics (age and forearm circumference) are provided as aggregate average values to avoid possible identification of participants.
Reporting on race, ethnicity, or other socially relevant groupings	No data on participant race, ethnicity, and other social groupings were collected.
Population characteristics	Data on participants age and forearm circumference was collected. Data is reported as aggregate average values to avoid the possibility of identification.
Recruitment	Participants were recruited by posters, via email (for participants who have previously participated in other studies, shared their contact details and confirmed that they are willing to receive invitations for further studies via email), and word of mouth. To minimize any potential experimenter bias participants were accepted on the study on a first come first served basis.
Ethics oversight	All procedures were approved in accordance with the declaration of Helsinki by the Imperial College Research Ethics Committee (ref: 22IC7602).

Note that full information on the approval of the study protocol must also be provided in the manuscript.

Field-specific reporting

Please select the one below that is the best fit for your research. If you are not sure, read the appropriate sections before making your selection.

[x] Life sciences [ ] Behavioural & social sciences [ ] Ecological, evolutionary & environmental sciences

For a reference copy of the document with all sections, see nature.com/documents/nr-reporting-summary-flat.pdf

Life sciences study design

All studies must disclose on these points even when the disclosure is negative.

Sample size	Ten participants were recruited for the offline sessions. Eight of the initial ten participants plus eight more were recruited for the online sessions. Two participants participated in the offline session but were not available to participate in the online sessions when asked. The recruitment sample size has been based on previous studies exploring ultrasound-based control studies. Indeed, recruited sizes match or are higher than previous studies (due to the long sessions the dataset size per participant of this study is considerably larger than others).<br><br>N. Akhlaghi et al., "Real-Time Classification of Hand Motions Using Ultrasound Imaging of Forearm Muscles," in IEEE Transactions on Biomedical Engineering, vol. 63, no. 8, pp. 1687-1698, Aug. 2016, doi: 10.1109/TBME.2015.2498124.<br><br>CC. Castellini and D. S. Gonzalez, "Ultrasound imaging as a human-machine interface in a realistic scenario," 2013 IEEE/RSJ International Conference on Intelligent Robots and Systems, Tokyo, Japan, 2013, pp. 1486-1492, doi: 10.1109/IROS.2013.6696545.<br><br>X. Yang, J. Yan, Z. Yin and H. Liu, "Sonomyographic Prosthetic Interaction: Online Simultaneous and Proportional Control of Wrist and Hand Motions Using Semisupervised Learning," in IEEE/ASME Transactions on Mechatronics, vol. 28, no. 2, pp. 804-813, April 2023, doi: 10.1109/TMECH.2022.3207359.

	B. G. Sgambato et al., "High Performance Wearable Ultrasound as a Human-Machine Interface for Wrist and Hand Kinematic Tracking," in IEEE Transactions on Biomedical Engineering, vol. 71, no. 2, pp. 484-493, Feb. 2024, doi: 10.1109/TBME.2023.3307952.
Data exclusions	Four participants were initially recruited and joined a pilot experiment. No data from these participants was used due to issues with the recording software and due to large changes in experimental protocol between them and the final cohort. No data from the ten participants on the final cohort was excluded from analysis.
Replication	Data collection from each participant was carried out independently. Ultrasound signal preprocessing and image formation parameters were the same for all participants. Network hyperparameters were only generally optimized to reasonable values but not individually optimized for any single cross-validation training sets. The same final model as used by all participants in the online evaluation session without any changes or calibration.
Randomization	A number of cross-validation strategies were employed to explore robustness in varied realistic scenarios, the complete details are available on the Data Collection sub-section. Briefly, data was evaluated in the SameSet scenario (standard approach used in the literature where each recording is split linearly into train/test), cross-functional scenario (without arm, elbow, and trunk movements in the training set), cross-positions (across armband positions along the forearm), cross-rotation (across armband rotations), cross-sessions (across both recording sessions of each participant), cross-participant (on new, never seem before, participants).
Blinding	During the offline experiment participants were encouraged to be relaxed and mimic the guidance videos but informed to no be worried about missing movements or doing wrong motions. Users were blind on how well they were performing the movements. During the online sessions no participants reported experience with VR environments featuring realistic physics, and over 60\% had little to no VR experience.

Reporting for specific materials, systems and methods

We require information from authors about some types of materials, experimental systems and methods used in many studies. Here, indicate whether each material, system or method listed is relevant to your study. If you are not sure if a list item applies to your research, read the appropriate section before selecting a response.

Materials & experimental systems

n/a	Involved in the study
☒	☐ Antibodies
☒	☐ Eukaryotic cell lines
☒	☐ Palaeontology and archaeology
☒	☐ Animals and other organisms
☒	☐ Clinical data
☒	☐ Dual use research of concern
☒	☐ Plants

Methods

n/a	Involved in the study
☒	☐ ChIP-seq
☒	☐ Flow cytometry
☒	☐ MRI-based neuroimaging

Plants

Seed stocks	n/a
Novel plant genotypes	n/a
Authentication	n/a
